# POEMS syndrome mimicking diabetic peripheral neuropathy: a case report and literature review

**DOI:** 10.3389/fmed.2026.1993357

**Published:** 2026-10-21

**Authors:** Yemei Chen, Chuxuan Fang, Shanfei Liang, Xinfei Xie, Shuangyi Tang, Zongxuan Huang

**Affiliations:** 1 The First Affiliated Hospital of Guangxi Medical University, Nanning, China; 2 The Second People’s Hospital of Nanning, Nanning, China; 3 Guangxi Medical University, Nanning, China

**Keywords:** bortezomib, daratumumab, diabetic peripheral neuropathy, lenalidomide hypersensitivity, monoclonal gammopathy, peripheral neuropathy, POEMS syndrome, vascular endothelial growth factor

## Abstract

POEMS syndrome is a rare multisystem plasma cell disorder characterized by peripheral neuropathy and a broad spectrum of systemic manifestations. Because peripheral neuropathy may coexist with common metabolic disorders, POEMS syndrome can be easily overlooked or misdiagnosed. We report the case of a 64-year-old woman with a 2-year history of diabetes mellitus who presented with progressive numbness of all four limbs for more than 11 months and fatigue for approximately 1 year. She was initially diagnosed with diabetic peripheral neuropathy and treated with glucose-lowering and neuroprotective therapy, but her neurological symptoms persisted and progressed. Further evaluation demonstrated demyelinating peripheral neuropathy with axonal involvement, an IgA-*λ* monoclonal protein, a clonal plasma cell population in the bone marrow, markedly elevated plasma vascular endothelial growth factor (VEGF), bilateral papilledema, lymphadenopathy, lower-extremity edema, and characteristic skin changes. Based on the combination of mandatory, major, and minor diagnostic features, POEMS syndrome was diagnosed. The patient initially received lenalidomide plus dexamethasone (RD), but lenalidomide was discontinued after the development of a generalized inflammatory erythematous rash with scaling and pruritus during the second treatment cycle. Treatment was subsequently changed to daratumumab, bortezomib, and dexamethasone (Dara + VD). During Dara + VD treatment, serum M-protein became undetectable, plasma VEGF decreased from 842.29 pg/mL before treatment to 836 pg/mL after the first cycle, and edema, fatigue, abdominal distension, and peripheral neuropathic symptoms improved. However, the patient was discharged after receiving only the first administration of the fourth treatment cycle. Three days after discharge, she developed generalized seizures with loss of consciousness and subsequently experienced severe systemic complications, including pneumonia, sepsis, septic shock, renal failure, and heart failure. She eventually died of multiple organ dysfunction syndrome. This case highlights the diagnostic challenge of POEMS syndrome in patients with pre-existing diabetes mellitus. Progressive or atypical peripheral neuropathy that is disproportionate to the presumed metabolic disorder should prompt evaluation for monoclonal plasma cell disorders and POEMS syndrome, particularly when accompanied by edema, skin changes, lymphadenopathy, papilledema, or elevated VEGF. In this patient, Dara + VD was associated with a short-term hematological and clinical response after lenalidomide intolerance; however, the subsequent fatal course precludes conclusions regarding its long-term efficacy.

## 1 Introduction

POEMS syndrome is a rare multisystem disorder associated with a monoclonal plasma cell disorder and characterized by polyneuropathy, organomegaly, endocrinopathy, monoclonal protein, and skin changes. The clinical manifestations extend beyond the features represented by the acronym and may include extravascular volume overload, papilledema, osteosclerotic bone lesions, lymphadenopathy, thrombocytosis or erythrocytosis, and other systemic abnormalities (1–4).

The rarity and multisystem nature of POEMS syndrome frequently result in delayed diagnosis. Peripheral neuropathy is one of the most common initial manifestations and may resemble chronic inflammatory demyelinating polyradiculoneuropathy (CIDP) or other peripheral neuropathies (3, 5). The diagnostic challenge becomes particularly pronounced when patients have pre-existing diabetes mellitus, because diabetic peripheral neuropathy provides a common and apparently plausible explanation for sensory symptoms. However, progressive neuropathy, demyelinating electrophysiological abnormalities, or neurological symptoms accompanied by systemic manifestations should prompt reconsideration of the initial diagnosis.

Here, we report a 64-year-old woman with pre-existing diabetes mellitus who developed progressive four-limb numbness and fatigue and was initially diagnosed with diabetic peripheral neuropathy. Subsequent evaluation revealed a monoclonal plasma cell disorder, markedly elevated VEGF, demyelinating peripheral neuropathy, edema, skin changes, lymphadenopathy, and papilledema, leading to the diagnosis of POEMS syndrome. The patient subsequently developed suspected lenalidomide hypersensitivity and was treated with Dara + VD, with a short-term hematological and clinical response followed by severe systemic complications and death. This case illustrates the importance of recognizing POEMS syndrome in patients whose peripheral neuropathy is not adequately explained by common metabolic disorders.

## 2 Case presentation

### 2.1 Patient information and clinical presentation

A 64-year-old woman was admitted to our hospital with numbness of all four limbs for more than 11 months and fatigue for approximately 1 year.

Approximately 1 year before admission, the patient developed dizziness and fatigue without an obvious precipitating factor. She was evaluated at a local hospital and was initially diagnosed with type 2 diabetes mellitus-associated peripheral neuropathy. She subsequently received symptomatic treatment, including neurotrophic therapy and glucose-lowering therapy. Her symptoms temporarily improved, and she was discharged.

Approximately 1 month after discharge, she developed arthralgia, purple discoloration of both hands upon exposure to cold temperatures, recurrent dizziness, and progressive numbness of the limbs. She subsequently attended the neurology outpatient clinic of our hospital.

Nerve conduction studies demonstrated abnormalities in motor and sensory nerve conduction in all four limbs, abnormal F-wave responses, and findings suggestive of multiple peripheral nerve and nerve root involvement, with combined demyelinating and axonal injury. Sympathetic skin response testing also demonstrated abnormalities involving the bilateral median nerves and cervical nerves.

The patient was treated with oral epalrestat and mecobalamin; however, her neurological symptoms did not significantly improve. Because of persistent and progressive symptoms despite treatment for presumed diabetic peripheral neuropathy, she was admitted for further evaluation.

At admission, the patient reported poor sleep quality but remained in a relatively stable general condition. Her appetite, urination, and bowel movements were normal, and she reported no significant recent weight change.

### 2.2 Medical history and physical examination

The patient had a 2-year history of diabetes mellitus and was receiving regular treatment with glimepiride. She also had a 1-year history of hypothyroidism and was receiving levothyroxine replacement therapy.

She denied smoking and alcohol consumption and had no known history of hereditary disease. She had a history of allergy to penicillin antibiotics. Ten years earlier, she had undergone surgery involving the left upper lobe of the lung at our hospital; however, detailed information regarding the indication and operative procedure was unavailable.

On admission, her temperature was 36.4 °C, pulse rate was 80 beats/min, respiratory rate was 19 breaths/min, and blood pressure was 116/56 mmHg. Cardiac, pulmonary, and abdominal examinations revealed no obvious abnormalities. Mild bilateral lower-extremity pitting edema was present.

Neurological examination showed preserved muscle strength (Medical Research Council grade 5/5) in all extremities, normal muscle tone, and no pathological reflexes.

### 2.3 Diagnostic evaluation

A comprehensive laboratory and auxiliary examination was performed after admission. The principal findings are summarized in Table 1.

**Table 1:** Principal laboratory and auxiliary examination findings on admission.

Category	Findings
Hematology	Hemoglobin, 109 g/L
Liver/nutritional status	Total protein, 48.5 g/L; albumin, 29.5 g/L
Renal function	Urea, 10.60 mmol/L; uric acid, 422 μmol/L; creatinine clearance, 30.9 mL/min
Serum protein electrophoresis	IgA-*λ* monoclonal protein
Plasma VEGF	894.29 pg/mL (reference limit, ≤96.2 pg/mL)
Endocrine/metabolic assessment	T3, 1.02 nmol/L; free T3, 2.89 pmol/L; 25-hydroxyvitamin D3, 17.92 nmol/L; parathyroid hormone, 10.12 pmol/L; sex hormones, 24-h urinary free cortisol, and ACTH without reported abnormalities
Bone marrow smear	Markedly active trilineage hematopoiesis; plasma cells approximately 8%, with a proliferative pattern
Bone marrow biopsy/immunohistochemistry	CD38 and CD138 positive in scattered/small clusters; sparse *κ*-positive cells and scattered/small clusters of λ-positive cells
Electrophysiology	Multiple peripheral nerve and nerve-root involvement with combined demyelinating and axonal injury; abnormal sympathetic skin responses
Superficial organ ultrasonography	Enlarged lymph nodes; focal thyroid lesion
Ophthalmological examination	Bilateral papilledema
Skin examination	Hyperpigmentation and livid discoloration; bilateral lower-extremity pitting edema; no generalized rash or subcutaneous bleeding; no palmar erythema or spider angiomas

The plasma VEGF concentration was 894.29 pg/mL, approximately 9.3-fold higher than the upper reference limit of 96.2 pg/mL. Electrophysiological examination demonstrated peripheral neuropathy with both demyelinating and axonal features. Bilateral papilledema was identified on ophthalmological examination.

Bone marrow examination demonstrated hypercellular marrow with trilineage hematopoiesis and increased plasma cells. Bone marrow smear showed plasma cells accounting for approximately 8%, with a proliferative pattern. Immunohistochemical staining showed scattered and clustered CD38- and CD138-positive plasma cells with predominant *λ* light-chain restriction, supporting a clonal plasma cell disorder.

The patient also had generalized hyperpigmentation, livid skin changes, bilateral lower-extremity pitting edema, and enlarged superficial lymph nodes. Representative bone marrow aspirate morphology is shown in Figure 1.

**Figure 1:**
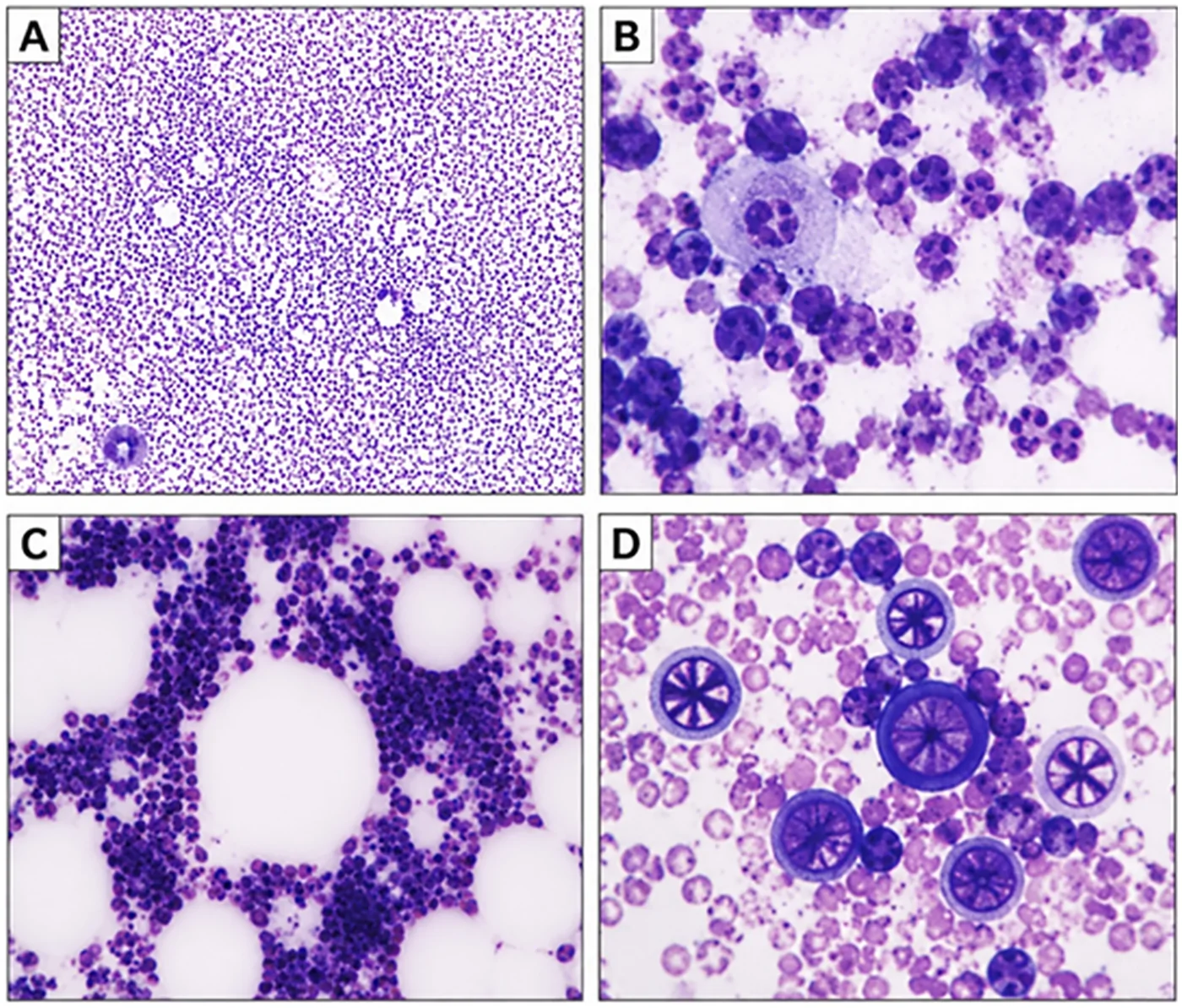
Bone marrow aspirate smear morphology. **(A–D)** Representative microscopic findings.

Electrophysiological examination demonstrated abnormalities involving the motor and sensory nerves of all four limbs, with abnormal F-wave responses. The findings indicated involvement of multiple peripheral nerves and nerve roots, with both demyelinating and axonal features. Sympathetic skin responses were also qualitatively abnormal. The principal documented findings are summarized in Table 2.

**Table 2:** Principal documented nerve-conduction study findings.

Parameter	Nerve	Documented finding	Available detail
Motor nerve conduction	All four limbs	Abnormal	Numerical latency/amplitude/conduction velocity values were not retained in the available clinical record
Sensory nerve conduction	All four limbs	Abnormal	Numerical values not available in the source record
F-wave responses	All four limbs	Abnormal	Numerical values not available in the source record
Localization	Multiple peripheral nerves and nerve roots	Abnormal	Combined peripheral nerve and nerve-root involvement documented
Electrophysiological pattern	Peripheral neuropathy	Demyelinating + axonal	Demyelinating injury with concomitant axonal injury
Sympathetic skin response	Bilateral median nerves and cervical nerves	Abnormal	Qualitative abnormality documented

The original clinical record available for this report did not contain numerical latency, amplitude, or conduction-velocity values for individual nerves. To avoid introducing unsupported data, the revised table reports only the electrophysiological parameters and qualitative findings explicitly documented in the source record.

### 2.4 Comprehensive diagnostic work-up and diagnostic reasoning

The diagnosis of POEMS syndrome was established based on the presence of both mandatory diagnostic features together with at least one major criterion and at least one minor criterion. The complete diagnostic work-up is summarized in Table 3.

**Table 3:** Comprehensive diagnostic work-up for POEMS syndrome.

Diagnostic domain	Criterion	Status	Evidence in the present case
Polyneuropathy	Typically demyelinating peripheral neuropathy	Present	Four-limb motor and sensory abnormalities, abnormal F waves, and combined demyelinating and axonal injury
Monoclonal plasma cell disorder	Usually lambda light-chain restricted; confirmed by bone marrow and serum/urine studies	Present	IgA-λ monoclonal protein; approximately 8% plasma cells with λ-predominant immunophenotype
Castleman disease	Histological confirmation	Not assessed	Enlarged superficial lymph nodes were present, but no Castleman-specific histology was documented
Sclerotic bone lesions	Osteosclerotic bone lesions on imaging	Not assessed	No definitive osteosclerotic bone lesion was documented in the available case record.
Elevated VEGF	Increased plasma VEGF	Present	VEGF 894.29 pg/mL (reference limit, ≤96.2 pg/mL)
Organomegaly	Hepatomegaly, splenomegaly, or lymphadenopathy	Present	Enlarged superficial/axillary lymph nodes on ultrasonography
Extravascular volume overload	Peripheral edema, pleural effusion, ascites, or pericardial effusion	Present	Bilateral lower-extremity pitting edema
Endocrinopathy	Abnormal adrenal, thyroid, pituitary, gonadal, parathyroid, or pancreatic function	Present, but attribution to POEMS not established	Pre-existing diabetes mellitus and hypothyroidism; their temporal relationship to POEMS was not established
Skin changes	Hyperpigmentation, hypertrichosis, hemangiomas, erythema, cyanosis, flushing, white nails	Present	Hyperpigmentation and livid discoloration
Papilledema	Bilateral optic disc edema	Present	Bilateral papilledema
Thrombocytosis/erythrocytosis	Elevated platelet count and/or hemoglobin	Not assessed	Platelet count was not available in the case record; hemoglobin was 109 g/L
Other characteristic features	Clubbing, weight loss, hyperhidrosis, pulmonary hypertension, restrictive lung disease, thrombosis	Not assessed systematically	No complete structured assessment was documented

The patient fulfilled both mandatory criteria: polyneuropathy and a monoclonal plasma cell disorder with IgA-*λ* restriction. The plasma VEGF concentration was 894.29 pg/mL, approximately 9.3-fold higher than the reference limit of 96.2 pg/mL, fulfilling a major criterion. Several minor features were also present, including lymphadenopathy, extravascular volume overload manifested by bilateral lower-extremity edema, characteristic skin changes, and bilateral papilledema. Although diabetes mellitus and hypothyroidism were present, their temporal relationship to the multisystem syndrome could not be established; therefore, these conditions were not considered sufficient to account for the overall clinical presentation. The diagnosis of POEMS syndrome was made based on the integrated clinical, electrophysiological, laboratory, pathological, and ophthalmological findings.

### 2.5 CARE-style clinical timeline

The clinical course, including the major diagnostic and therapeutic events and the subsequent clinical outcome, is summarized in the CARE-style timeline in Table 4.

**Table 4:** CARE-style timeline of the patient's clinical course.

Time point	Clinical event	Clinical significance
Approximately 1 year before admission	Dizziness and fatigue developed	Initially considered diabetic peripheral neuropathy
After initial treatment	Glucose-lowering and neurotrophic therapy	Temporary symptomatic improvement
Approximately 1 month after discharge	Recurrent dizziness and limb numbness	Neurological reassessment initiated
Neurology evaluation	Nerve conduction testing	Demyelinating and axonal peripheral neuropathy identified
Subsequent evaluation	Multisystem diagnostic work-up	POEMS-related features identified
After diagnostic work-up	POEMS syndrome diagnosed	Plasma cell-directed therapy initiated
Initial systemic treatment	RD regimen	Lenalidomide discontinued because of drug-induced dermatitis
Subsequent treatment	Dara + VD regimen	Clinical and hematological improvement observed
After the first administration of cycle 4	Discharged after first administration	Cycle 4 was not completed
Three days after discharge	Generalized seizures with loss of consciousness	Readmitted for further treatment
Subsequent hospitalization	Severe systemic complications	Progressive systemic deterioration
Final outcome	Multiple organ dysfunction syndrome	Death

### 2.6 Treatment and clinical course

Before the diagnosis of POEMS syndrome was established, the patient received symptomatic and supportive treatment, including epalrestat, vitamin B1, mecobalamin, glucose-lowering therapy with glimepiride, levothyroxine replacement, and other supportive interventions. She also received traditional Chinese medicine-based treatments, electroacupuncture, intermediate-frequency therapy, and acupoint application. These interventions did not result in substantial improvement in her neurological symptoms.

After transfer to the Department of Hematology and confirmation of POEMS syndrome, plasma cell-directed therapy was initiated with lenalidomide plus dexamethasone (RD). Lenalidomide was administered at 25 mg/day on days 1–21, and dexamethasone at 40 mg/day on days 1, 8, 15, and 22. The patient's creatinine clearance was 30.9 mL/min; according to the treating physician, lenalidomide dosing was adjusted with consideration of renal function. During the second treatment cycle, the patient developed generalized inflammatory erythema, particularly involving the trunk and extremities, accompanied by scaling and pruritus. No lesions were observed in the oral cavity or external genitalia. Drug-induced dermatitis was diagnosed, and lenalidomide was discontinued, after which the rash improved.

The treatment regimen was subsequently changed to daratumumab, bortezomib, and dexamethasone (Dara + VD). Daratumumab 800 mg was administered intravenously on days 1, 8, 15, and 22, with the dose determined based on the patient's body weight; bortezomib 1.5 mg was administered subcutaneously on days 2, 9, 16, and 23, with the dose determined according to the patient's clinical condition rather than calculated solely on the basis of body surface area; and dexamethasone 10 mg was administered on days 2, 9, 16, and 23.

During Dara + VD treatment, the patient's bilateral lower-extremity edema gradually improved. Fatigue and abdominal distension also improved progressively, while peripheral numbness showed gradual improvement, with only minimal residual symptoms by the third cycle. A hematological response was also observed. Before Dara + VD, serum M-protein was 0.93 g/L and plasma VEGF was 842.29 pg/mL. After the first cycle, M-protein became undetectable, although IgA-*λ* remained positive and plasma VEGF remained elevated at 836 pg/mL. No additional VEGF measurements were available in the retained records; therefore, the available data do not establish a sustained downward trend in VEGF. The treatment course and documented clinical and hematological responses are summarized in Table 5.

**Table 5:** Treatment course and documented clinical and hematological responses.

Phase	Treatment	Clinical response	Hematological response
Baseline before Dara + VD	—	Persistent bilateral lower-extremity numbness and edema	IgA-λ positive; M-protein 0.93 g/L; plasma VEGF 842.29 pg/mL
Cycle 1	Dara + VD	Lower-extremity edema partially improved; numbness persisted	M-protein became undetectable; IgA-λ remained positive; plasma VEGF 836 pg/mL
Cycle 2	Dara + VD	Further improvement in edema, abdominal distension, and fatigue	M-protein remained undetectable; globulin abnormalities gradually improved
Cycle 3	Dara + VD	Marked improvement in fatigue and abdominal distension; minimal residual numbness	IgA and λ light-chain signals weakened; M-protein remained undetectable
Cycle 4 (first administration only)	Dara + VD	No significant treatment-related adverse events were documented before discharge	Hematological response remained present based on available assessments

### 2.7 Clinical outcome

After the first administration of the fourth treatment cycle, the patient had no clinically significant acute treatment-related adverse events and was discharged from the hospital.

Three days after discharge, however, she developed generalized convulsions accompanied by loss of consciousness and was readmitted. During the subsequent hospitalization, she developed severe pneumonia, sepsis, septic shock, renal failure, heart failure, and status epilepticus. Despite comprehensive supportive treatment and management of the associated neurological and systemic complications, her condition progressively deteriorated. She eventually died of multiple organ dysfunction syndrome.

The short-term response observed during Dara + VD treatment therefore did not translate into a favorable overall clinical outcome in this patient.

## 3 Discussion

### 3.1 Diagnostic considerations in POEMS syndrome

POEMS syndrome is a rare multisystem plasma cell disorder characterized by peripheral neuropathy, a monoclonal plasma cell disorder, and a heterogeneous combination of organomegaly, endocrinopathy, skin changes, extravascular volume overload, papilledema, and elevated vascular endothelial growth factor (VEGF) (1–5). Because these manifestations are heterogeneous and may overlap with more common neurological disorders, POEMS syndrome can be overlooked, particularly when peripheral neuropathy is the predominant presentation (5–8).

The major diagnostic consideration in patients with progressive neuropathy is whether the neurological findings can adequately explain the overall clinical phenotype. Diabetic peripheral neuropathy is common and may readily be assumed in patients with diabetes mellitus. However, progressive involvement of multiple limbs, prominent demyelinating abnormalities, or neurological deterioration disproportionate to the underlying diabetes should prompt reconsideration of alternative diagnoses. POEMS-associated neuropathy may show demyelinating features accompanied by substantial axonal loss and can therefore overlap electrophysiologically with chronic inflammatory demyelinating polyneuropathy (CIDP) (6–8). Electrophysiological findings should consequently be interpreted together with hematological and systemic findings rather than used in isolation.

In this context, the detection of a monoclonal plasma cell disorder is particularly important. POEMS is generally associated with a relatively small plasma cell clone, most commonly with lambda light-chain restriction (3, 4, 9). Therefore, a modest plasma cell burden should not be regarded as evidence against POEMS when other characteristic features are present. Similarly, individual systemic manifestations such as edema, skin pigmentation, lymphadenopathy, endocrine abnormalities, or papilledema may be nonspecific. Their diagnostic significance increases substantially when they occur together with polyneuropathy and a monoclonal plasma cell disorder. Thus, the diagnosis of POEMS requires a multisystem approach rather than reliance on a single clinical manifestation or laboratory abnormality (2–4, 9–11).

### 3.2 Diagnostic value of VEGF and multisystem assessment

VEGF has an important role in the pathophysiology of POEMS syndrome and is also a valuable diagnostic and disease-monitoring biomarker (3, 12, 13). Increased VEGF is associated with vascular permeability and extravascular volume overload and may help distinguish POEMS from other causes of demyelinating polyneuropathy. In the present case, the plasma VEGF concentration was 894.29 pg/mL, approximately 9.3-fold higher than the upper reference limit of 96.2 pg/mL for the assay used. This marked elevation, together with the monoclonal IgA-*λ* protein and multisystem manifestations, provided important evidence supporting the diagnosis.

The diagnostic value of VEGF should nevertheless be considered in conjunction with the established diagnostic framework rather than as an isolated screening test. A comprehensive evaluation should include neurological assessment, monoclonal protein studies, bone marrow examination, VEGF measurement, endocrine evaluation, assessment of volume overload and skin changes, ophthalmological examination, and appropriate imaging (2–4, 9–11). This integrated approach is particularly important in patients whose initial presentation resembles a common neurological or metabolic disorder.

Another important consideration is that the underlying plasma cell clone in POEMS is often small compared with that observed in overt multiple myeloma (3, 4, 9). Therefore, the absence of extensive plasma cell proliferation should not delay diagnosis when the characteristic clinical constellation is present. Conversely, findings such as lymphadenopathy or bone abnormalities should be interpreted according to their specific clinical and imaging characteristics rather than automatically being regarded as diagnostic of POEMS. Overall, this case illustrates that early recognition depends on identifying the coherence among neurological, hematological, vascular, endocrine, dermatological, and ophthalmological abnormalities.

### 3.3 Therapeutic considerations and treatment tolerance

The principal therapeutic goal in POEMS syndrome is to suppress the underlying plasma cell clone and thereby reduce the biological abnormalities associated with the disease, particularly VEGF-mediated vascular dysfunction (3, 4, 9). Treatment selection should be individualized according to disease distribution, plasma cell burden, neurological involvement, bone lesions, comorbidities, renal function, and eligibility for autologous stem cell transplantation. Localized disease may be considered for radiotherapy, whereas systemic therapy is generally required when disease is disseminated or associated with substantial systemic involvement (3, 4, 10, 11).

Lenalidomide plus dexamethasone is an established systemic treatment option and has been associated with hematological, VEGF, and neurological responses in previous studies (14, 15). However, treatment tolerance is an important consideration in individual patients. In the present case, lenalidomide was discontinued after the development of generalized inflammatory erythema, particularly involving the trunk and extremities, with scaling and pruritus. No lesions were observed in the oral cavity or external genitalia, and drug-induced dermatitis was diagnosed. The rash subsequently improved after discontinuation of lenalidomide. The patient's creatinine clearance was 30.9 mL/min, and according to the treating physician, lenalidomide dosing was adjusted with consideration of renal function. These findings emphasize the need for individualized assessment of adverse reactions and organ function when selecting and maintaining systemic therapy.

Daratumumab-containing regimens have increasingly been explored as therapeutic options for POEMS syndrome, particularly in patients who are not able to tolerate or respond adequately to conventional approaches (16, 17). In this case, daratumumab combined with bortezomib and dexamethasone (Dara + VD) was associated with short-term clinical and hematological improvement, including disappearance of detectable M-protein and improvement in edema, fatigue, abdominal distension, and neuropathic symptoms. However, only two VEGF measurements were available during this treatment period, decreasing from 842.29 pg/mL before Dara + VD to 836 pg/mL after cycle 1. Therefore, these data should be interpreted as an observed decrease rather than evidence of a sustained VEGF response. The available clinical literature on daratumumab-containing regimens in POEMS remains limited, and the therapeutic efficacy of this approach requires further evaluation in larger studies (16, 17). In the present case, the short-term response suggests potential clinical activity of Dara + VD after intolerance to lenalidomide, but the subsequent severe systemic complications and fatal outcome preclude conclusions regarding its long-term efficacy.

### 3.4 Clinical implications and interpretation of the outcome

An important lesson from this case is that improvement in hematological or selected clinical parameters does not necessarily indicate complete disease control or guarantee a favorable long-term outcome. The subsequent severe neurological and systemic complications occurred after the patient had received only the first administration of the fourth treatment cycle. Because multiple complications developed during the subsequent hospitalization, including severe infection, septic shock, renal failure, heart failure, and status epilepticus, the final fatal outcome should not be interpreted simply as treatment failure or attributed to a single disease mechanism.

For clinicians, the key implication is the need for longitudinal and multidisciplinary management of POEMS syndrome. Neurological symptoms, monoclonal protein, VEGF, volume status, endocrine abnormalities, ophthalmological findings, infection risk, organ function, and treatment tolerance should be assessed dynamically. In patients with diabetes mellitus and progressive peripheral neuropathy, the emergence of atypical electrophysiological findings or otherwise unexplained systemic abnormalities should prompt reassessment for POEMS syndrome. Early recognition may facilitate appropriate plasma cell-directed treatment before irreversible neurological or systemic complications develop (2–4, 10, 11).

## 4 Conclusion

POEMS syndrome is a rare multisystem plasma cell disorder that may be difficult to recognize when peripheral neuropathy is initially attributed to a common metabolic disorder such as diabetes mellitus. In the present case, progressive four-limb numbness persisted despite conventional treatment for presumed diabetic peripheral neuropathy. The subsequent identification of demyelinating peripheral neuropathy, an IgA-*λ* monoclonal plasma cell disorder, markedly elevated VEGF, lymphadenopathy, edema, skin changes, and papilledema established the diagnosis of POEMS syndrome. This case emphasizes that clinicians should reconsider the diagnosis when neuropathy is progressive, atypical, or disproportionate to the underlying metabolic disease. After intolerance to lenalidomide-based therapy, Dara + VD was associated with short-term hematological and clinical improvement; however, the subsequent development of severe systemic complications and death underscores the complexity of this disorder and precludes conclusions regarding long-term treatment efficacy.

## Data Availability

The author selected the following statement: The datasets presented in this study can be found in online repositories. The names of the repository/repositories and accession number(s) can be found in the article/supplementary material. And I did not detect any particular expressions.
